# Genetic Evidence for the Association between Schizophrenia and Breast Cancer

**DOI:** 10.20900/jpbs.20180007

**Published:** 2018-08-08

**Authors:** Jiajun Shi, Lang Wu, Wei Zheng, Wanqing Wen, Shuyang Wang, Xiang Shu, Jirong Long, Chen-Yang Shen, Pei-Ei Wu, Emmanouil Saloustros, Jenny Chang-Claude, Hermann Brenner, Xiao-Ou Shu, Qiuyin Cai

**Affiliations:** 1 Department of Medicine, Division of Epidemiology, Vanderbilt Epidemiology Center, Vanderbilt-Ingram Cancer Center, Vanderbilt University School of Medicine, Nashville, TN 37203, USA;; 2 Institute of Biomedical Sciences, Academia Sinica, Taipei 11529, Taiwan;; 3 Taiwan Biobank, Academia Sinica, Taipei 11529, Taiwan;; 4 University hospital of Larisa, Larisa, 41110, Greece;; 5 Division of Cancer Epidemiology, German Cancer Research Center (DKFZ), Heidelberg 69120, Germany;; 6 University Cancer Center Hamburg (UCCH), University Medical Center Hamburg-Eppendorf, Hamburg 20246, Germany;; 7 Division of Clinical Epidemiology and Aging Research, German Cancer Research Center (DKFZ), Heidelberg 69120, Germany;; 8 German Cancer Consortium (DKTK), German Cancer Research Center (DKFZ), Heidelberg 69120, Germany;; 9 Division of Preventive Oncology, German Cancer Research Center (DKFZ) and National Center for Tumor Diseases (NCT), Heidelberg 69120, Germany.

**Keywords:** breast cancer, schizophrenia, Mendelian randomization, instrumental variables, genome-wide association study

## Abstract

**Objective:**

To estimate the potential effect of schizophrenia on breast cancer risk in women, we performed a two-sample Mendelian randomization (MR) study.

**Methods:**

The instrumental variables comprised 170 uncorrelated and non-pleiotropic single nucleotide polymorphisms (SNPs) that are significantly associated with schizophrenia risk in genome-wide association studies in 105,000 European descent individuals of the Psychiatric Genomics Consortium (http://www.med.unc.edu/pgc/) and the United Kingdom Clozapine Clinic. The association between these SNPs determined schizophrenia and breast cancer risk was estimated in approximately 229,000 European descent females from the Breast Cancer Association Consortium using the inverse-variance weighted and the weighted median MR methods.

**Results:**

We found that the genetically-predicted risk of schizophrenia was associated with increased breast cancer risk (under a random-effects model: odds ratio per 1 unit increase in log odds of schizophrenia = 1.04, 95% confidence interval: 1.02–1.06, *p* = 5.6 × 10^−5^). Similar significant associations were observed in analyses using a weighted median model and sensitivity analysis excluding six SNPs with genotype imputation score of less than 0.8, as well as analyses stratified by estrogen receptor status of breast cancer.

**Conclusion:**

Our findings implicate a modest increased risk for breast cancer in genetically determined schizophrenic females.

## 1 INTRODUCTION

Observational studies have suggested an increased breast cancer risk in female subjects with schizophrenia (SCZ) ^[1–3]^. Antipsychotic-induced hyperprolactinemia has been suggested as a risk factor for increased breast cancer risk in SCZ women, while other known breast cancer risk factors, including nulliparity, obesity, type-2 diabetes, alcohol dependence, smoking, and low physical activity, are more likely to be the cause of comorbidity ^[4]^. However, it is unknown whether there is a genetic effect of SCZ on breast cancer risk.

Mendelian randomization (MR), a design that utilizes genetic variants as instrumental variables (IVs), could potentially be used to estimate the unconfounded effect of an exposure/risk factor on an outcome ^[5]^. Compared to traditional epidemiologic methods, MR is less prone to confounding effects due to the random assortment of alleles at conception. Recent large-scale genome-wide association studies (GWAS) have identified multiple genetic variants associated with complex human traits or diseases including SCZ ^[6,7]^ and breast cancer ^[8–10]^, which enable MR analysis by using such genetic variants as IVs with an increased statistical power to detect potential causal associations of exposure with an outcome ^[11–13]^. Two-sample MR has become popular, as it exploits publicly available summary data of genetic instrument-exposure association and genetic instrument-outcome association in GWAS consortia from different samples of participants ^[14,15]^.

To address potentially biased association between SCZ and breast cancer risk due to unmeasured confounders, we conducted a two-sample MR study by analyzing publicly accessible summary meta-analysis results of two SCZ GWAS data sets, the Psychiatric Genomics Consortium (PGC2) ^[6]^ and the United Kingdom Clozapine Clinic (CLOZUK) ^[7]^, and one breast cancer GWAS data set from the Breast Cancer Association Consortium(BCAC,http://apps.ccge.medschl.cam.ac.uk/consortia/bcac/) ^[10]^. These studies represent the largest sample size GWAS to date for each of the diseases in European descendants. The genetic effect of SCZ on breast cancer was further evaluated by estrogen receptor (ER) status of the cancer tissues.

## 2 MATERIALS AND METHODS

**Fig. 1:**
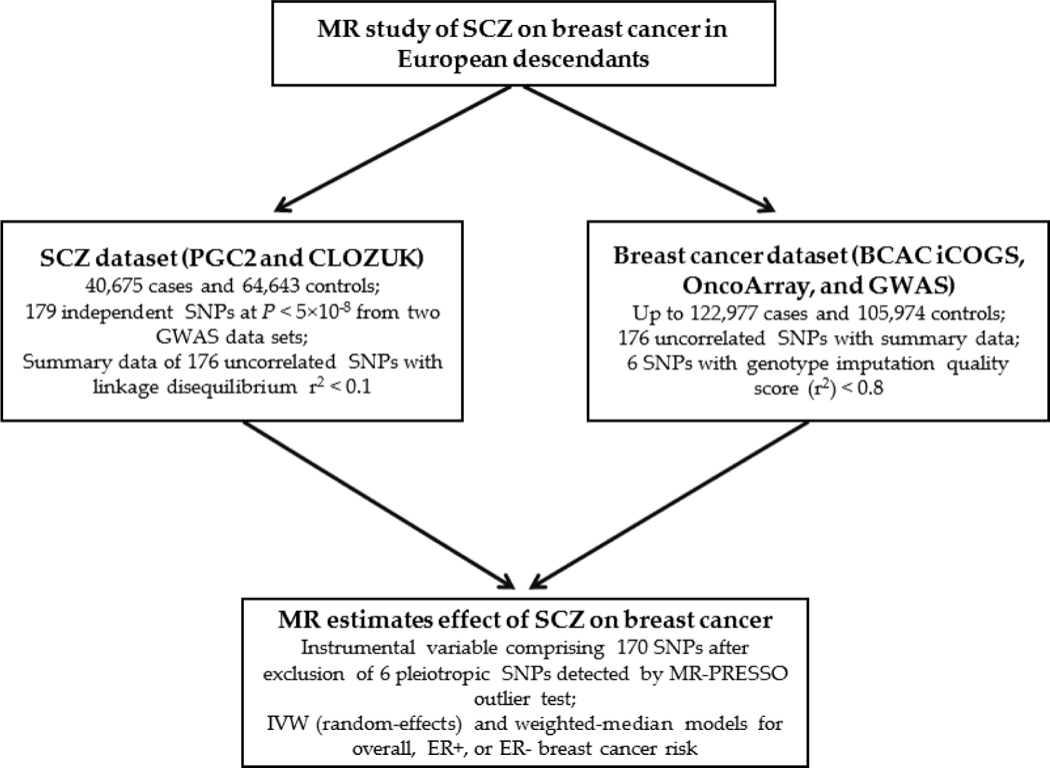
Flowchart depicting current Mendelian randomization analyses of effect of schizophrenia on breast cancer. The details of the genome-wide association studies from the Psychiatric Genomics Consortium (PGC2) and the United Kingdom Clozapine Clinic (CLOZUK) for SCZ and the Breast Cancer Association Consortium (BCAC) were previously described ^[7,10]^.

Fig. 1 shows the overall design of our study regarding the process of selecting genetic instruments in the two-sample MR, the sources of summary genetic association data and the statistical models used.

### 2.1 GWAS datasets for MR

Single nucleotide polymorphisms (SNPs) significantly associated with SCZ risk were identified from the most recent largest-scale GWAS combing association results of PGC2 and CLOZUK ^[6,7]^. Summary associations of these SNPs with breast cancer risk were obtained from the latest GWAS by the BCAC ^[10]^.

In the SCZ GWAS comprising 40,675 cases and 64,643 controls of European descendants, 179 common SNPs (minor allele frequency (MAF) > 0.01) at 145 distinct genomic loci were identified to be significantly associated with disease risk (*p* < 5 × 10^−8^) ^[7]^. The summary association data for each of the 179 SNPs were downloaded from the Data Repository website (http://walters.psycm.cf.acuk/) of the Walters Group at the Cardiff University MRC Centre for Neuropsychiatric Genetics and Genomics. The sex-combined association summary statistics was selected as few sex-specific associated genetic variants have been reported ^[16]^, and there are no suggested sex differences in SCZ prevalence ^[17]^. To reduce potential violation of MR assumptions due to linkage disequilibrium (LD) of SNPs at a single locus ^[18]^, independent SNPs with LD *r*^*2*^ of less than 0.1 were selected based on the genotypic data of 503 individuals of European ancestry from the 1000 Genomes Project phase 3 dataset. A total of 176 SNPs remained after removing three SNPs (rs66791238, rs199687649, and rs67439964) with *r*^2^ > 0.1 with another more significant index SNP located nearby (Supplementary Table S1).

The latest BCAC GWAS included 122,977 breast cancer cases and 105,974 controls of European ancestry from three datasets with different study designs and genotyping platforms (the OncoArray (http://bcac.ccge.medschl.cam.ac.uk/bcacdata/oncoarray/): 61,282 cases and 45,494 controls; the Collaborative Oncological Gene-Environment Study (iCOGS, http://ccge.medschlcam.ac.uk/research/consortia/icogs/ ): 46,785 cases and 42,892 controls; and 11 other breast cancer GWAS: 14,910 cases and 17,588 controls) ^[10]^. For the 176 uncorrelated SCZ associated SNPs, summary breast cancer association data were retrieved from combined samples from the BCAC database (http://bcac.ccge.medschl.cam.ac.uk/bcacdata/oncoarray/gwas-icogs-and-oncoarray-summary-results/ ). To reduce distorted effects of genetic IVs, six horizontally pleiotropic SNPs (rs7632921, rs16902086, rs3130820, rs10650434, rs2905432, and rs17514846) identified from the Mendelian randomization pleiotropy residual sum and outlier (MR-PRESSO, https://githubcom/rondolab/MR-PRESSO ) test ^[19]^ were further removed. Finally, a total of 170 SNPs were selected to estimate the effect of genetically determined SCZ on breast cancer risk. MR was also performed after excluding six SNPs with imputation quality score *r*^2^ of less than 0.8 in the BACA controls. Characteristics and the summary association statistics of each of the SNPs are provided in Supplementary Table S1.

### 2.2 Statistical analysis

Summary statistical data of SNP-SCZ association were first standardized with the effect allele of each SNP to be associated with increased SCZ risk. The corresponding dataset of SNP-breast cancer association were then harmonized through matching the effect alleles to be consistent with those in the exposure dataset. The SNP-exposure and SNP-outcome association datasets were then combined using the inverse-variance weighted (IVW) method ^[20]^. This approach is based on the assumption that SNP-outcome associations are entirely mediated through the exposure factor, with the intercept of pleiotropic effect constrained at zero ^[12]^. The resulting estimate effect of the exposure on the outcome is equal to the coefficient from a weighted regression of SNP-outcome on SNP-exposure association estimate, i.e., a random-effects meta-analysis of the ratio estimates from each SNP.

As previously described for the IVW method ^[20]^, let *x* and *y* denote the exposure and outcome, respectively. The parameter *α* was used to quantify the causal effect of *x* on *y*. Let *γ*_i_ and *β*_i_ denote effect-size estimates of the *i*th SNP on *x* and *y*, respectively, and let *se* (*ß*i) denote the standard error (*s.e*) of *β*_i_. Then the MR estimate associated with the ith SNP is *α*_i_ = *β*_*i*_∕*γ*_*i*_*,* and the corresponding variance of this estimate is1 $v_{\text{i}}={\left(s.e\cdot \left(\beta_{\text{i}}\right)/\gamma_{\text{i}}\right)}^{2}$ *.* The weight of the *i*th MR estimate of *α* is defined as $w_{\text{i}}=1/v_{\text{i}}$. The IVW random-effects estimate is $\alpha_{\text{random}}=\sum_{i=1}^{n}\alpha_{i}w_{\text{i}}/\sum_{i=1}^{n}w_{\text{i}}$ and the *s.e.* of the estimate is given by $s.e.={\left(\sum_{i=1}^{n}w_{\text{i}}\right)}^{-1/2}$. A random-effects model was used in this study because multiple SNPs were included and the heterogeneity of effect size among these SNPs is most likely to exist. Cochran’s *Q* statistical analysis was used to test heterogeneity and the *I* ^2^ statistic was used to estimate the amount of heterogeneity ^[21]^.

SCZ-breast cancer effect was also estimated using a weighted median method which allows up to 50% of genetic instruments to be invalid ^[22]^. Finally, further MR was conducted to test the effect of SCZ on risk of ER-positive and ER-negative breast cancer.

The effect-sizes for each meta-analysis are reported as the odds ratios (ORs) describing the effect of SCZ on breast cancer risk (per genetically predicted 1-unit-higher log-odds of SCZ). A *p* < 0.05 was used to define statistical significance. All the MR analyses were conducted using the MR-PRESSO and MR-Base (http://www.mrbase.org/) “TwoSampleMR” packages ^[23]^ in R version 3.4.3 (http://www.r-project.org/).

## 3 RESULTS

Using the 170 SCZ-associated SNPs as instrumental variables, a significant association between genetically-predicted SCZ risk and risk of breast cancer was observed in women of European ancestry through the random-effects IVW MR (OR per 1 unit increase in log odds of SCZ: 1.04; 95% CI: 1.02–1.06; *p* = 5.6 × 10^−5^) (Table 1). Similar overall breast cancer risk association estimates were obtained using the weighted median model (Table 1), as well as the sensitivity analysis excluding six SNPs with an imputation *r*^2^ < 0.8 (Table 2).

When breast cancer was stratified by ER status, significant associations of genetically predicted SCZ risk were detected with both ER-positive breast cancer and ER-negative breast cancer risk using the random-effects IVW or the weighted median models, with ORs ranging from 1.03 to 1.05 (Table 1). When six SNPs with an imputation *r*^2^ of < 0.8 were excluded, the identified associations remained for both ER-positive (IVW: OR =1.04; 95% CI: 1.02–1.07) and ER-negative breast cancer (IVW: OR = 1.04; 95% CI: 1.01–1.07) (Table 2).

## 4 DISCUSSION

In this large-scale MR study in European female descendants, we estimated genetic influence of 170 independent non-pleiotropic SCZ-associated SNPs on breast cancer risk. Results from both the standard IVW random-effects and the weighted median models suggest a positive association between genetically determined SCZ and breast cancer risk. The association was also detected in both ER-positive and ER-negative breast cancer.

A meta-analysis of 16 observational studies in 427,843 patients with SCZ showed a 25% increased co-occurrence of breast cancer ^[2]^. The most recent meta-analysis of 12 cohort studies with 125,760 female SCZ patients revealed a 31% increased breast cancer risk, although significant heterogeneity between studies existed ^[3]^. Antipsychotic-induced hyperprolactinemia, nulliparity, obesity, type-2 diabetes and unhealthy lifestyle behaviors (e.g., smoking, alcohol dependence, and low physical activity) have been proposed for breast cancer risk factors in female patients with SCZ ^[4]^; however, it is unknown what proportion of breast cancer risk variation is explained by these non-genetic factors. On the other hand, two recent studies suggested a nominally significant positive genetic correlation between SCZ and breast cancer (*r* = 0.14–0.16) ^[24,25]^. The horizontal pleiotropy of some genetic variants may account for this genetic association ^[20]^. In this study, we excluded six pleiotropic SNPs through MR-PRESSO outlier tests and still detected a significant weak genetic association. The modest effect from our analyses may be true, due to the low incidence of breast cancer in schizophrenic females ^[1]^, the low genetic correlation between these two diseases ^[24,25]^, and the low proportion (29%) of genetic component of SCZ influencing breast cancer risk ^[25]^.

Our study has several strengths. First, this two-sample MR study, using publicly accessible summary statistics from the largest-scale SCZ GWAS ^[7]^ and breast cancer GWAS ^[10]^, found a significant genetic influence of SCZ on breast cancer risk. Second, the influence of SCZ on breast cancer risk was observed for both ER-positive and ER-negative diseases (Tables 1 and 2), showing their possible common etiology link to genetically predicted SCZ. Third, results from the IVW random-effects and the weighted median models support a reliable estimate. Fourth, we excluded pleiotropic SNPs which potentially distorted the estimate in MR analyses.

There are also some potential limitations in the present study. First, as SCZ is a binary exposure, the estimated effect on breast cancer risk from the random-effects IVW MR may still be biased ^[26]^, although significant pleiotropic SNPs have been excluded. A recently developed robust method, named as “MR G-Estimation under No Interaction with Unmeasured Selection”, can provide valid inferences for the average causal effect of binary exposure on binary outcome. However, this method requires individual level genotype data and externally estimated parameters for the underlying population in the context of case-control studies ^[27]^. Second, the SNP-SCZ associations were based on analyses of combined sex and not for women only, and thus, potential population stratification may exist. However, population stratification (including that caused by sex difference) in each of the GWAS was controlled using principal components during SNP-SCZ association analyses ^[6,7]^. Third, the underlying biological mechanisms of increased breast cancer risk in female SCZ patients remain unclear. A previously proposed hypothesis of antipsychotic-induced hyperprolactinemia as the cause for breast cancer has been shown to be inconclusive since hyperprolactinemia has also been observed in antipsychotic-naïve first-episode patients and even in prodromal stages, and several prolactin-elevating antipsychotics have been shown to have cancer-protection mechanisms ^[4]^. On the other hand, enrichment of cell and tissue type-specific enhancers of SCZ-associated SNPs support a role for immune dysregulation ^[6]^, while a similar dysfunctional immune system hypothesis has been proposed for the pathogenesis of breast cancer ^[28]^. Further studies are warranted to clarify whether the SCZ-related immune system ^[6]^ or other biological mechanism(s) contribute to the development of breast cancer. Fourth, invalid or weak genetic instrumental variables may introduce biased effect in MR analyses since many of the SCZ GWAS-identified SNPs with association *p* < 5 × 10^−8^ from GWAS need to be further replicated in independent samples to avoid the winner’s curse bias or inflated effect sizes. For example, among 108 SCZ-associated loci from PGC2, 15 have not reached genome-wide significance in the combined PGC2 and CLOZUK samples ^[7]^. Fifth, results from the heterogeneity tests suggest a possible horizontal/ pleiotropic effect of the SCZ-associated SNPs, which could influence the effect size estimate. However, we excluded SNPs that are pleiotropic outlier SNPs using MR-PRESSO. In addition, LD score regression ^[29]^ with approximately 1,700 uncorrelated SNPs (*r*^2^ < 0.1) across the genome that were associated with SCZ at *p* < 1.0 × 10^−4^ in the PGC2 European participants ruled out a global pleiotropism between SCZ and breast cancer (posterior probability < 1%). Sixth, clinically observed increased incidence of breast cancer in female SCZ patients may be a result of surveillance bias. In other words, a closer clinical care of SCZ patients than other patients would possibly lead to an earlier diagnosis of breast cancer. MR approaches may not be able to deal with such bias. Seventh, it is unknown whether the MR detected SCZ-breast cancer association in European descendants could be generalizable to population of other ancestry. Lastly, MR analyses using genetic risk score method with individual genotype data and detailed breast cancer risk factors, such as nulliparity, obesity, type-2 diabetes, smoking, alcohol dependence, and low physical activity, are needed to clarify the genetic effect of SCZ on breast cancer.

## 5 CONCLUSION

Genetically determined schizophrenic females may have a modest increased risk for breast cancer.

## Supplementary Material

supplemental file

## Figures and Tables

**Table 1.** Mendelian randomization estimates of the effect of SCZ on breast cancer risk in European descendants.

Breast cancer	Method	OR (95% CI) ^a^	*p* ^a^	*p*_het^b^	*I*^2^^b^
Overall (122,977 cases and 105,974 controls)					

	IVW-random	1.04 (1.02–1.06)	5.6 × 10^−5^	2.3 × 10^−10^	45%
	Weighted Median	1.03 (1.01–1.06)	9.2 × 10^−3^	-	-

ER-positive (69,501 cases and 95,042 controls)					

	IVW-random	1.04 (1.02–1.07)	2.2 × 10^−4^	4.2 × 10^−7^	39%
	Weighted Median	1.03 (1.01–1.06)	0.019	-	-

ER-negative (21,468 cases and 100,594 controls)					

	IVW-random	1.04 (1.01–1.07)	7.2 × 10^−3^	0.032	17%
	Weighted Median	1.05 (1.01–1.09)	0.026	-	-

Abbreviations: SCZ: schizophrenia; OR: odds ratio; CI: confidence interval; IVW, inverse-variance weighted.

a OR estimates of SCZ on breast cancer based on the random-effects IVW or the weighted-median MR.

b Heterogeneity test for causal ratio estimates of all 170 selected genetic instrumental variables.

**Table 2.** Mendelian randomization estimates of the effect of SCZ on breast cancer risk using single nucleotide polymorphisms with imputation quality score (*r*^2^) > 0.8 in breast cancer controls.

Breast cancer	Method	OR (95% CI)a	*p* ^a^	*p*_het^b^	*I*^2^^b^
Overall (122,977 cases and 105,974 controls)					

	IVW-random	1.04 (1.02–1.06)	1.7 × 10^−4^	2.5 × 10^−10^	46%
	Weighted Median	1.03 (1.01–1.06)	9.8 × 10^−3^	-	-

ER-positive (69,501 cases and 95,042 controls)					

	IVW-random	1.04 (1.02–1.07)	4.4 × 10^−4^	2.6 × 10^−7^	40%
	Weighted Median	1.04 (1.01–1.06)	0.021	-	-

ER-negative (21,468 cases and 100,594 controls)					

	IVW-random	1.04 (1.01–1.07)	0.016	0.028	18%
	Weighted Median	1.04 (0.99–1.08)	0.076	-	-

Abbreviations: SCZ: schizophrenia; OR: odds ratio; CI: confidence interval; IVW, inverse-variance weighted.

a OR estimates of SCZ on breast cancer based on the random-effects IVW or the weighted-median MR.

b Heterogeneity test for causal ratio estimates of 164 genetic instrumental variables with genotype imputation score > 0.8.
