# Supplementary material for: Genetic Evidence for the Association between Schizophrenia and Breast Cancer
Source: J Psychiatr Brain Sci. Author manuscript; Available in PMC 2019 Mar 6. (PMC6402491; doi:10.20900/jpbs.20180007)
Supplement: supplemental file [file NIHMS1001247-supplement-supplemental_file.pdf]

JPBS 2018; 3(4): 7; https://doi.org/10.20900/jpbs.20180007

Supplementary Materials: Genetic Evidence for the Association between Schizophrenia and Breast Cancer

Jiajun Shi[1], Lang Wu[1], Wei Zheng[1], Wanqing Wen[1], Shuyang Wang[1], Xiang Shu[1], Jirong Long[1], Chen-Yang Shen[2], Pei-Ei Wu[3], Emmanouil Saloustros[4], Jenny Chang-Claude[5,6], Hermann Brenner[7,8,9], Xiao-Ou Shu[1], Qiuyin Cai[1]*

1 Division of Epidemiology, Department of Medicine, Vanderbilt Epidemiology Center, Vanderbilt-Ingram Cancer Center, Vanderbilt University School of Medicine, Nashville, TN 37203, USA;

2 Institute of Biomedical Sciences, Academia Sinica, Taipei 11529, Taiwan;

3 Taiwan Biobank, Academia Sinica, Taipei 11529, Taiwan;

4 University hospital of Larisa, Larisa, 41110, Greece;

5 Division of Cancer Epidemiology, German Cancer Research Center (DKFZ), Heidelberg 69120, Germany;

6 University Cancer Center Hamburg (UCCH), University Medical Center Hamburg-Eppendorf, Hamburg 20246, Germany;

7 Division of Clinical Epidemiology and Aging Research, German Cancer Research Center (DKFZ), Heidelberg 69120, Germany;

8 German Cancer Consortium (DKTK), German Cancer Research Center (DKFZ), Heidelberg 69120, Germany;

9 Division of Preventive Oncology, German Cancer Research Center (DKFZ) and National Center for Tumor Diseases (NCT), Heidelberg 69120, Germany.

*Corresponding Author: Qiuyin Cai, Email: qiuyin.cai@vanderbilt.edu.

Table S1. Characteristics of selected SCZ-associated SNPs in the current Mendelian randomization of SCZ on breast cancer risk.

SNP	Chr	Base position_b37	Effect allele	Other allele	Effect allele frequency[a]	BCAC_ onco2_ Imputation_r^{2b}	OR_SCZ	*p*_SCZ	OR_BC	*p*_BC	Note
rs4648845	1	2387101	T	C	0.48	0.95	1.08	6.74×10^{-12}	0.99	0.2103	Selected for MR
rs34269918	1	8424984	ga	g	0.68	0.99	1.06	3.27×10^{-9}	1.01	0.3695	Selected for MR
rs6694545	1	30437268	A	G	0.24	0.97	1.08	6.20×10^{-12}	1.01	0.4889	Selected for MR
rs2970610	1	44097530	T	C	0.36	1	1.07	1.39×10^{-11}	0.99	0.08941	Selected for MR
rs12129719	1	66324512	A	G	0.56	0.97	1.06	3.35×10^{-08}	0.99	0.3098	Selected for MR
rs12129573	1	73768366	A	C	0.40	1	1.08	8.94×10^{-15}	1.00	0.7473	Selected for MR

Table S1. ***Cont.***

SNP	Chr	Base position_b37	Effect allele	Other allele	Effect allele frequency[a]	BCAC_ onco2_ Imputation_r^{2b}	OR_SCZ	*p*_SCZ	OR_BC	*p*_BC	Note
rs6680011	1	95840866	C	A	0.15	1	1.08	2.83×10^{-8}	1.00	0.8881	Selected for MR
rs11165867	1	97878068	T	C	0.16	1	1.07	3.87×10^{-8}	1.00	0.8983	Selected for MR
rs2660304	1	98512127	T	G	0.79	0.99	1.11	2.18×10^{-18}	1.00	0.854	Selected for MR
rs140505938	1	150031490	C	T	0.83	1	1.09	6.50×10^{-10}	1.00	0.7292	Selected for MR
rs6701877	1	174015259	G	T	0.82	0.99	1.08	2.37×10^{-8}	0.99	0.4819	Selected for MR
rs4650963	1	177309490	T	G	0.14	0.95	1.09	1.16×10^{-08}	0.99	0.3678	Selected for MR
rs55770408	1	190643601	C	T	0.24	0.99	1.06	3.02×10^{-8}	0.99	0.2365	Selected for MR
rs28374258	1	190949551	A	T	0.19	1	1.08	6.35×10^{-10}	0.99	0.3069	Selected for MR
rs6678676	1	200266503	C	T	0.78	0.99	1.07	3.22×10^{-8}	0.99	0.1815	Selected for MR
rs72769124	1	239210058	A	C	0.10	1	1.11	4.73×10^{-10}	1.01	0.3946	Selected for MR
rs10803138	1	243555219	G	A	0.73	0.99	1.07	2.03×10^{-09}	0.98	0.02217	Selected for MR
rs14403	1	243663893	C	T	0.79	0.99	1.08	1.71×10^{-10}	1.00	0.7403	Selected for MR
rs12712510	2	22749726	T	C	0.47	0.98	1.06	8.18×10^{-9}	1.00	0.7339	Selected for MR
rs77011057	2	58064341	A	G	0.94	0.99	1.12	4.48×10^{-8}	1.00	0.7771	Selected for MR
rs75575209	2	58138192	T	A	0.08	0.99	1.1	4.60×10^{-9}	1.03	0.008013	Selected for MR
rs7596038	2	58383820	C	T	0.56	1	1.07	2.37×10^{-12}	1.00	0.4509	Selected for MR
rs2077586	2	73161551	A	G	0.73	0.71	1.06	2.96×10^{-8}	1.00	0.8398	Selected for MR
rs56145559	2	73623439	T	C	0.21	0.99	1.07	1.01×10^{-9}	1.00	0.901	Selected for MR
rs12991836	2	145141541	C	A	0.37	0.79	1.06	6.46×10^{-10}	1.01	0.1097	Selected for MR
rs56807175	2	146436222	TC	T	0.18	0.98	1.09	1.36×10^{-11}	1.01	0.36	Selected for MR
rs10196799	2	185640728	A	T	0.58	0.98	1.06	4.51×10^{-9}	1.00	0.9907	Selected for MR
rs6434928	2	198304577	G	A	0.27	1	1.08	3.62×10^{-13}	1.01	0.4184	Selected for MR
rs1451488	2	199990107	G	A	0.53	0.98	1.07	4.75×10^{-12}	1.03	4.45×10^{-5}	Selected for MR
rs34719143	2	200244409	T	TC	0.94	0.77	1.09	7.71×10^{-9}	1.04	0.005777	Selected for MR

Table S1. ***Cont.***

SNP	Chr	Base position_b37	Effect allele	Other allele	Effect allele frequency[a]	BCAC_ onco2_ Imputation_r^{2b}	OR_SCZ	*p*_SCZ	OR_BC	*p*_BC	Note
rs76432012	2	200576767	T	C	0.95	0.94	1.15	2.75×10^{-8}	1.04	0.006521	Selected for MR
rs2949006	2	200715388	T	G	0.18	0.99	1.11	3.69×10^{-17}	1.01	0.07467	Selected for MR
rs200626410	2	200768453	ttc	t	0.77	0.67	1.08	2.95×10^{-9}	1.01	0.3326	Selected for MR
rs1347692	2	201297861	C	T	0.47	0.98	1.06	4.11×10^{-10}	1.01	0.03389	Selected for MR
rs11685299	2	225391296	C	A	0.67	1	1.06	3.86×10^{-9}	1.01	0.1302	Selected for MR
rs4144797	2	233562197	T	C	0.39	0.97	1.09	4.33×10^{-16}	1.01	0.07592	Selected for MR
rs35346733	3	2521322	A	G	0.19	0.93	1.09	2.42×10^{-12}	1.00	0.9622	Selected for MR
rs6800435	3	10804551	A	C	0.12	0.98	1.09	2.00×10^{-8}	1.01	0.3573	Selected for MR
rs9881798	3	16846967	C	A	0.39	0.98	1.06	2.81×10^{-8}	0.99	0.2024	Selected for MR
rs11409090	3	17887635	AT	A	0.48	0.96	1.06	2.11×10^{-9}	1.02	0.00998	Selected for MR
rs75968099	3	36858583	T	C	0.36	1	1.07	9.41×10^{-11}	0.99	0.1599	Selected for MR
rs1080500	3	53175017	G	A	0.67	0.88	1.08	2.71×10^{-12}	1.01	0.3137	Selected for MR
rs312477	3	53515136	G	A	0.77	1	1.07	1.38×10^{-8}	1.00	0.862	Selected for MR
rs1353545	3	60287845	C	G	0.34	0.99	1.06	5.67×10^{-9}	0.99	0.2905	Selected for MR
rs704373	3	63867355	A	G	0.36	1	1.07	1.39×10^{-10}	1.03	9.76×10^{-5}	Selected for MR
rs7632921	3	71543758	G	T	0.56	0.99	1.06	9.52×10^{-9}	1.03	9.24×10^{-6}	Excluded due to pleiotropy by MR-PRESSO
rs7432375	3	136288405	G	A	0.56	1	1.07	4.07×10^{-12}	1.02	0.01898	Selected for MR
rs489939	3	161470592	G	A	0.63	1	1.06	1.24×10^{-8}	1.01	0.3329	Selected for MR
rs34796896	3	180623255	G	A	0.79	1	1.09	3.19×10^{-12}	1.00	0.7559	Selected for MR
rs55672338	3	181051857	A	T	0.52	0.97	1.06	1.52×10^{-9}	0.99	0.1033	Selected for MR
rs215411	4	23423603	A	T	0.31	1	1.06	1.40×10^{-8}	0.99	0.1179	Selected for MR
rs13107325	4	103188709	T	C	0.08	1	1.17	1.19×10^{-16}	1.02	0.1657	Selected for MR

Table S1. ***Cont.***

SNP	Chr	Base position_b37	Effect allele	Other allele	Effect allele frequency[a]	BCAC_ onco2_ Imputation_r^{2b}	OR_SC Z	*p*_SCZ	OR_BC	*p*_BC	Note
rs13121251	4	143829759	T	C	0.66	0.99	1.06	4.06×10^{-8}	1.01	0.3866	Selected for MR
rs10520163	4	170626552	T	C	0.50	1	1.05	2.81×10^{-8}	1.00	0.9727	Selected for MR
rs12498839	4	176731401	A	G	0.04	0.97	1.14	9.67×10^{-11}	1.01	0.3557	Selected for MR
rs62334820	4	176855221	T	C	0.19	0.97	1.08	9.60×10^{-12}	1.02	0.02595	Selected for MR
rs16902086	5	45285752	G	A	0.36	1	1.07	5.55×10^{-11}	1.05	8.19×10^{-12}	Excluded due to pleiotropy by MR-PRESSO
rs77853293	5	49840113	C	T	0.45	1	1.06	1.77×10^{-8}	1.03	5.26×10^{-5}	Selected for MR
rs7701440	5	60620980	C	T	0.47	0.99	1.08	3.72×10^{-14}	1.00	0.7998	Selected for MR
rs254782	5	88000630	G	A	0.96	0.98	1.13	4.40×10^{-8}	1.00	0.927	Selected for MR
rs16867576	5	88746331	A	G	0.89	0.93	1.11	1.65×10^{-11}	1.00	0.8769	Selected for MR
rs13169274	5	137855305	C	T	0.50	0.99	1.06	7.06×10^{-10}	1.01	0.08214	Selected for MR
rs79212538	5	151993104	T	G	0.04	0.98	1.15	5.55×10^{-10}	1.02	0.224	Selected for MR
rs111294930	5	152177121	A	G	0.72	0.97	1.09	9.04×10^{-12}	1.00	0.8294	Selected for MR
rs2910032	5	152540354	C	T	0.46	1	1.07	3.72×10^{-11}	0.99	0.1082	Selected for MR
rs12522290	5	152797656	C	G	0.82	0.98	1.08	1.34×10^{-9}	1.00	0.8804	Selected for MR
rs3130820	6	29206683	T	A	0.93	1	1.28	2.12×10^{-44}	1.07	7.41×10^{-9}	Excluded due to pleiotropy by MR-PRESSO
rs1339227	6	73155701	C	T	0.66	0.98	1.06	3.76×10^{-10}	1.01	0.02659	Selected for MR
rs4470825	6	83789798	G	A	0.58	0.98	1.06	8.94×10^{-9}	1.00	0.7167	Selected for MR
rs217287	6	84407466	C	T	0.56	0.98	1.07	9.53×10^{-13}	1.01	0.4495	Selected for MR
rs634940	6	93077500	T	G	0.24	0.96	1.07	1.30×10^{-8}	1.01	0.2842	Selected for MR
rs760608	6	114719447	G	A	0.27	0.97	1.06	1.90×10^{-8}	1.01	0.06045	Selected for MR

Table S1. *Cont.*

SNP	Chr	Base position_b37	Effect allele	Other allele	Effect allele frequency[a]	BCAC_ onco2_ Imputation_r^{2b}	OR_SCZ	*p*_SCZ	OR_BC	*p*_BC	Note
rs35736453	6	128329493	tc	t	0.72	0.98	1.06	2.95×10^{-8}	1.01	0.1124	Selected for MR
rs72342102	6	143650817	t	ttttg	0.20	0.99	1.07	7.93×10^{-9}	1.00	0.829	Selected for MR
rs10650434	7	2025096	ACT	A	0.42	1	1.09	1.10×10^{-18}	0.98	0.000997	Excluded due to pleiotropy by MR-PRESSO
rs146678232	7	24777089	C	CA	0.17	0.99	1.07	1.63×10^{-8}	1.01	0.3398	Selected for MR
rs12704290	7	86427626	G	A	0.87	0.99	1.12	3.57×10^{-14}	1.01	0.3342	Selected for MR
rs147922658	7	86775498	taag	t	0.97	1	1.18	7.71×10^{-10}	0.98	0.2485	Selected for MR
rs7789569	7	104927586	T	C	0.36	1	1.07	7.00×10^{-11}	1.00	0.7235	Selected for MR
rs211829	7	110048893	T	C	0.58	1	1.06	2.29×10^{-9}	1.01	0.2826	Selected for MR
rs12705761	7	110976264	G	C	0.58	0.98	1.07	5.11×10^{-11}	1.01	0.2164	Selected for MR
rs7801375	7	131567263	G	A	0.85	0.99	1.08	6.27×10^{-9}	0.99	0.2387	Selected for MR
rs4523180	7	131619693	T	G	0.92	0.74	1.11	3.83×10^{-8}	1.01	0.5433	Selected for MR
rs3735025	7	137074844	T	C	0.64	0.94	1.07	7.02×10^{-11}	1.00	0.5676	Selected for MR
rs139425113	8	4180611	GA	G	0.22	1	1.07	8.48×10^{-9}	1.02	0.06371	Selected for MR
rs11993663	8	10032894	A	C	0.31	1	1.06	3.40×10^{-8}	0.99	0.3463	Selected for MR
rs2410572	8	18421474	G	A	0.43	0.99	1.06	1.07×10^{-8}	1.00	0.4823	Selected for MR
rs1042992	8	26269191	T	C	0.17	1	1.08	3.67×10^{-9}	1.00	0.9737	Selected for MR
rs2565065	8	27328021	A	G	0.28	1	1.07	1.74×10^{-9}	1.00	0.7532	Selected for MR
rs11783093	8	27425349	C	T	0.85	1	1.1	7.64×10^{-12}	1.03	0.000913	Selected for MR
rs55669358	8	34312412	C	T	0.11	1	1.1	1.37×10^{-8}	0.98	0.1345	Selected for MR
rs10156310	8	38209129	A	T	0.76	1	1.08	5.56×10^{-10}	0.99	0.2748	Selected for MR
rs1473594	8	60696526	T	C	0.40	1	1.07	3.33×10^{-11}	1.00	0.5711	Selected for MR
rs7010876	8	89264751	T	A	0.28	0.99	1.06	6.51×10^{-9}	1.00	0.7146	Selected for MR

Table S1. *Cont.*

SNP	Chr	Base position_b37	Effect allele	Other allele	Effect allele frequency[a]	BCAC_ onco2_ Imputation_r^{2b}	OR_SCZ	*p*_SCZ	OR_BC	*p*_BC	Note
rs36043959	8	111472014	A	G	0.19	1	1.08	4.07×10^{-12}	0.99	0.3556	Selected for MR
rs4976967	8	143293307	G	A	0.68	0.92	1.07	9.07×10^{-9}	0.99	0.4649	Selected for MR
rs58033671	8	143320118	acccct gcacgc ccg	a	0.55	0.96	1.09	8.65×10^{-18}	1.00	0.5138	Selected for MR
rs67439964	8	143343398	T	C	0.77	0.93	1.08	1.16×10^{-9}	1.01	0.4704	Excluded due to LD r^2 of 0.12 with rs58033671
rs1319017	9	84736303	A	G	0.35	0.98	1.07	7.82×10^{-11}	1.00	0.8014	Selected for MR
rs10985817	9	101071090	C	T	0.17	1	1.08	1.02×10^{-9}	1.01	0.1416	Selected for MR
rs7099380	10	18549016	A	G	0.56	1	1.06	1.47×10^{-8}	1.00	0.7617	Selected for MR
rs7893279	10	18745105	T	G	0.89	0.98	1.12	4.80×10^{-13}	1.00	0.7161	Selected for MR
rs7476192	10	104649375	T	G	0.65	0.91	1.08	3.77×10^{-12}	0.99	0.03129	Selected for MR
rs12416331	10	104928914	T	A	0.91	1	1.16	7.09×10^{-18}	1.00	0.7426	Selected for MR
rs1899543	11	24406419	T	A	0.55	1	1.06	1.23×10^{-9}	0.99	0.4091	Selected for MR
rs1765142	11	30378559	A	C	0.66	0.97	1.06	1.13×10^{-8}	1.01	0.06988	Selected for MR
rs7951870	11	46373311	C	T	0.18	0.98	1.1	2.99×10^{-13}	1.02	0.02453	Selected for MR
rs7129727	11	57484660	A	G	0.31	1	1.06	1.47×10^{-9}	1.02	0.008475	Selected for MR
rs58950470	11	65383755	T	G	0.34	0.99	1.06	2.07×10^{-8}	1.02	0.002873	Selected for MR
rs2514218	11	113392994	C	T	0.65	0.97	1.08	2.42×10^{-12}	1.01	0.3752	Selected for MR
rs4936277	11	113431960	A	G	0.58	0.98	1.06	1.52×10^{-8}	1.01	0.3049	Selected for MR
rs12293670	11	124612932	A	G	0.65	0.99	1.08	1.70×10^{-15}	1.00	0.567	Selected for MR
rs35774874	11	130811356	T	C	0.49	0.99	1.07	1.97×10^{-11}	1.00	0.8366	Selected for MR
rs5795787	11	132397822	g	gtaa	0.52	0.99	1.06	2.69×10^{-8}	1.00	0.6731	Selected for MR

Table S1. *Cont.*

SNP	Chr	Base position_b37	Effect allele	Other allele	Effect allele frequency[a]	BCAC_ onco2_ Imputation_r^{2b}	OR_SCZ	p_SCZ	OR_BC	p_BC	Note
rs2917569	11	132568255	T	C	0.50	0.96	1.06	3.11×10^{-10}	1.00	0.9166	Selected for MR
rs4936215	11	133852684	A	G	0.78	0.89	1.1	5.32×10^{-14}	1.00	0.5885	Selected for MR
rs893949	11	134296384	C	T	0.47	0.83	1.05	2.98×10^{-8}	1.00	0.4782	Selected for MR
rs2007044	12	2344960	G	A	0.37	1	1.09	5.63×10^{-20}	1.03	6.04×10^{-5}	Selected for MR
rs12823424	12	2514112	A	G	0.70	0.97	1.07	2.28×10^{-9}	1.01	0.4576	Selected for MR
rs1120004	12	23633432	T	G	0.24	1	1.06	1.42×10^{-8}	1.01	0.3364	Selected for MR
rs10783624	12	39522907	C	A	0.34	0.98	1.06	5.44×10^{-9}	1.00	0.6665	Selected for MR
rs324015	12	57490100	C	T	0.75	1	1.07	1.42×10^{-10}	1.00	0.5405	Selected for MR
rs61937595	12	57682956	C	T	0.91	0.92	1.13	3.28×10^{-11}	1.04	0.003089	Selected for MR
rs4240748	12	92246786	G	C	0.63	0.99	1.06	2.15×10^{-8}	0.99	0.2305	Selected for MR
rs36104021	12	103361112	C	G	0.87	0.93	1.09	7.31×10^{-9}	1.00	0.8788	Selected for MR
rs4766428	12	110723245	T	C	0.45	0.98	1.08	2.68×10^{-14}	1.01	0.04506	Selected for MR
rs2851447	12	123665113	G	C	0.28	0.99	1.09	5.55×10^{-16}	0.99	0.1377	Selected for MR
rs9545047	13	79859456	A	C	0.61	0.99	1.06	1.15×10^{-8}	0.99	0.2565	Selected for MR
rs10148671	14	29469373	C	T	0.63	1	1.07	5.46×10^{-10}	1.01	0.4454	Selected for MR
rs1191551	14	30000405	T	G	0.22	0.98	1.08	4.12×10^{-10}	1.01	0.311	Selected for MR
rs199687649	14	30189985	ca	c	0.15	0.9	1.07	7.31×10^{-9}	0.99	0.4263	Excluded due to LD r^2 of 0.11 with rs1191551
rs34179565	14	33298731	C	CA	0.47	0.98	1.06	8.88×10^{-9}	0.99	0.2351	Selected for MR
rs150437760	14	59981768	A	C	0.94	1	1.13	4.58×10^{-8}	0.98	0.193	Selected for MR
rs2332700	14	72417326	C	G	0.25	0.95	1.07	1.52×10^{-10}	1.01	0.04485	Selected for MR
rs35604463	14	99712032	G	A	0.55	0.99	1.06	1.66×10^{-8}	0.99	0.07341	Selected for MR
rs10083370	14	104314182	G	A	0.38	0.99	1.08	3.44×10^{-14}	0.99	0.1313	Selected for MR

Table S1. ***Cont.***

SNP	Chr	Base position_b37	Effect allele	Other allele	Effect allele frequency[a]	BCAC_ onco2_ Imputation_r^{2b}	OR_SCZ	*p*_SCZ	OR_BC	*p*_BC	Note
rs80020004	14	104495116	T	C	0.10	0.92	1.09	1.85×10^{-8}	1.00	0.9596	Selected for MR
rs56282503	15	40566759	C	T	0.30	0.88	1.06	2.30×10^{-8}	1.00	0.6239	Selected for MR
rs281299	15	47686081	T	C	0.62	1	1.06	2.19×10^{-8}	1.02	0.003907	Selected for MR
rs12898315	15	61854003	A	G	0.57	1	1.06	2.51×10^{-9}	1.01	0.3566	Selected for MR
rs12148337	15	70589272	T	C	0.48	0.98	1.06	1.16×10^{-8}	1.01	0.3248	Selected for MR
rs3743078	15	78894759	G	C	0.76	1	1.08	3.11×10^{-12}	1.00	0.7168	Selected for MR
rs783540	15	83254708	G	A	0.42	1	1.06	8.45×10^{-10}	1.00	0.7613	Selected for MR
rs12908161	15	85207825	A	G	0.74	0.99	1.07	9.41×10^{-10}	1.00	0.8334	Selected for MR
rs17514846	15	91416550	C	A	0.54	1	1.07	2.55×10^{-12}	1.03	6.35×10^{-6}	Excluded due to pleiotropy by MR-PRESSO
rs12447542	16	7744180	A	G	0.14	0.9	1.09	1.44×10^{-8}	1.00	0.6457	Selected for MR
rs7191183	16	9900057	C	T	0.32	0.99	1.06	6.31×10^{-9}	1.00	0.6729	Selected for MR
rs7499750	16	13749265	A	C	0.26	0.99	1.07	4.24×10^{-10}	1.00	0.7846	Selected for MR
rs198160	16	24240725	G	T	0.50	0.91	1.06	4.88×10^{-8}	0.99	0.4324	Selected for MR
rs11646127	16	29966277	G	C	0.56	1	1.07	5.52×10^{-13}	1.00	0.9542	Selected for MR
rs42945	16	58545426	G	A	0.56	0.97	1.07	2.25×10^{-10}	0.99	0.1888	Selected for MR
rs17465671	16	63712719	C	A	0.49	0.94	1.06	4.14×10^{-9}	1.01	0.0468	Selected for MR
rs1975802	16	68285847	G	A	0.17	1	1.07	3.56×10^{-8}	1.02	0.0164	Selected for MR
rs2161711	16	71359066	A	G	0.82	0.99	1.07	4.22×10^{-8}	1.01	0.4783	Selected for MR
rs7216638	17	2156453	T	A	0.64	0.99	1.07	4.59×10^{-10}	0.98	0.009876	Selected for MR
rs4925114	17	17711270	A	G	0.40	0.96	1.06	2.64×10^{-8}	1.02	0.002487	Selected for MR
rs66885728	17	19014831	G	C	0.87	0.74	1.09	1.47×10^{-8}	1.00	0.9011	Selected for MR
rs7225476	17	78561603	A	G	0.56	1	1.05	4.86×10^{-8}	1.01	0.05844	Selected for MR

Table S1. *Cont.*

SNP	Chr	Base position_b37	Effect allele	Other allele	Effect allele frequency[a]	BCAC_ onco2_ Imputation_r^{2b}	OR_SCZ	p_SCZ	OR_BC	p_BC	Note
rs5825114	18	52749216	ga	g	0.61	1	1.08	5.03×10^{-14}	1.01	0.3052	Selected for MR
rs79926379	18	53009097	G	A	0.04	0.97	1.18	1.61×10^{-11}	1.03	0.05325	Selected for MR
rs66791238	18	53230650	T	C	0.78	0.97	1.07	6.41×10^{-9}	1.01	0.3576	Excluded due to LD r^2 of 0.11 with rs5825114
rs28758902	18	53408187	T	C	0.46	1	1.07	4.75×10^{-13}	1.00	0.6464	Selected for MR
rs144158419	18	53554733	C	T	0.92	0.98	1.14	5.03×10^{-13}	1.01	0.2415	Selected for MR
rs1789595	18	53775254	A	T	0.71	1	1.07	2.23×10^{-09}	1.00	0.6505	Selected for MR
rs56775891	18	77575613	T	C	0.25	0.99	1.07	2.03×10^{-9}	1.00	0.5678	Selected for MR
rs72986630	19	11849736	T	C	0.06	0.84	1.15	8.09×10^{-10}	0.98	0.1099	Selected for MR
rs2905432	19	19484295	G	A	0.33	1	1.07	6.62×10^{-12}	1.05	9.23×10^{-12}	Excluded due to pleiotropy by MR-PRESSO
rs2053079	19	30987423	G	A	0.26	0.99	1.07	1.82×10^{-10}	1.01	0.1038	Selected for MR
rs7508148	19	50152142	T	C	0.74	0.98	1.08	4.06×10^{-11}	1.00	0.8563	Selected for MR
rs6035706	20	20821005	G	A	0.30	0.99	1.06	7.24×10^{-9}	1.01	0.1148	Selected for MR
rs6065094	20	37453194	G	A	0.65	1	1.09	7.91×10^{-17}	1.00	0.5726	Selected for MR
rs9611177	22	39840130	C	T	0.61	0.9	1.06	3.84×10^{-8}	0.99	0.2749	Selected for MR
rs5757730	22	39967430	G	A	0.56	0.94	1.07	1.76×10^{-12}	1.00	0.6199	Selected for MR
rs4820386	22	40068051	T	C	0.45	0.99	1.06	4.07×10^{-10}	1.01	0.1182	Selected for MR
rs9607782	22	41587556	A	T	0.27	0.97	1.08	5.54×10^{-13}	1.00	0.5132	Selected for MR
rs1023497	22	42340508	C	G	0.80	1	1.08	2.04×10^{-11}	1.00	0.9377	Selected for MR
rs6002655	22	42603814	T	C	0.43	0.95	1.08	2.15×10^{-14}	0.99	0.1143	Selected for MR
rs12009217	23	5920882	G	A	0.13	1	1.07	1.78×10^{-10}	1.01	0.08249	Selected for MR
rs62606711	23	68377204	A	G	0.18	0.99	1.07	1.26×10^{-12}	1.00	0.6163	Selected for MR

[a] Effective allele frequency in the 1000 Genomes EUR super-population. [b] Linkage disequilibrium (LD) data are based on 1000 Genomes Phase 3 EUR.

© 2018 by the authors, Licensee Qingres, London, United Kingdom. This is an open access article distributed under the terms and conditions of the Creative Commons Attribution License (CC BY 4.0, https://creativecommons.org/licenses/by/4.0/).
